# Two Cases of Cervical Hemorrhage with Upper Airway Obstruction: A Life-Threatening Condition

**DOI:** 10.1155/2014/674176

**Published:** 2014-01-30

**Authors:** Enrico Maria Amadei, Laura Benedettini, Ottavio Piccin

**Affiliations:** ^1^Department of Otorhinolaryngology, Head and Neck Surgery, S. Orsola-Malpighi University Hospital, Via Pietro Albertoni 15, 40138 Bologna, Italy; ^2^Department of Transfusion Medicine and Immunohematology, Bufalini Hospital, Viale Ghirotti 286, 47521 Cesena, Italy

## Abstract

Several are the causes of cervical masses and among them a spontaneous hemorrhage presents a rare and life-threatening condition. Sometimes hemorrhage develops from a previous silent neck lesion as in the case of an anaplastic thyroid carcinoma associated with bleeding. We present two cases: a 70-year-old woman suffering from enlarging cervical mass causing respiratory distress because of upper airway compression due to a spontaneous rupture of the superior thyroid artery and a 74-year-old woman who drew our attention because of a progressively worsening dyspnea due to a large medial cervical mass with rapid onset. We removed it surgically, finding out an anaplastic thyroid carcinoma that is associated with internal bleeding. We discuss our management of these rare and life-threatening conditions, recalling that the patency of upper airway should always be the prerogative in every emergency. Besides, we make a review of the recent literature.

## 1. Introduction

A spontaneous cervical hemorrhage presents a rare and life-threatening condition due to potential airway injury. The ethiologies of spontaneous cervical hemorrhage may be different; according to Gonzàlez-Cruz et al. [1], the most plausible explanation of haematoma in multinodular goitre is venous bleeding due to an increasing blood supply. In spontaneous thyroid haemorrhage, an increase in venous pressure after a Valsalva manoeuvre was postulated; another cause is hemodynamic alteration in the context of a hemodialysis session, along with the use of heparin [2]. Saylam et al. [3] claim that secondary thyroid hemorrhage of a previously normal thyroid gland as a result of trauma is a very rare condition; possible causes of bleeding could be: trauma, deceleration injury, cervical hyperflexion and Valsalva manoeuvre which increases venous pressure [4], including straining during defecation or heavy lifting. In our opinion hypertension can be the trigger in patients with known or unknown thyroid disease [5], especially if patients present coagulopathy [6].

It is also possible that a sudden neck swelling occurs due to hemorrhage of a rare thyroid cancer, such as an anaplastic thyroid carcinoma (ATC). This is the rarest, but the deadliest histologic type among thyroid malignancies, with a dismal median survival of 3–9 months [7–9]. It represents less than 2% [7, 10] of all thyroid tumors; however, this is the cause of 14%–39% of thyroid carcinoma-related deaths. The female/male ratio is 5 to 1 and the peak of incidence is in the sixth and seventh decade of life. Usually, it turns out to be a rapidly growing cervical mass causing dyspnea, dysphagia, and vocal cord paralysis. It is extremely difficult to recognize it in its early stages. The therapeutic attempt is based both on a radical surgery and on radiotherapy or chemotherapy using doxorubicin and cisplatin [7–9].

We present two rare cases of airway compromission: a spontaneous intrathyroid hemorrhage due to the rupture of the superior thyroid artery (STA) and a bleeding ATC.

## 2. Case Report Number 1

A 70-year-old woman presented to our outpatient department with a sudden swelling on the left side of her neck. At the beginning, the patient did not show any dyspnea or dysphonia, but only enhancing dysphagia. Indirect laryngoscopy revealed dislocation of the larynx to the right side. She denied symptoms of an upper respiratory tract infection or history of trauma. Her medical history displayed assumption of Warfarin, because of a periodical deep venous thrombosis. She suffered from hypertension, which was pharmacologically well treated.

Ultrasound scan revealed a large neck hematoma with transversal diameters of 4.8 × 6.3 cm. Then, the increasing size of the swelling connected to early dyspnea aroused the suspicious of an acute cervical hemorrhage, hence the patient was subjected to an angiography.

**Figure 1:**
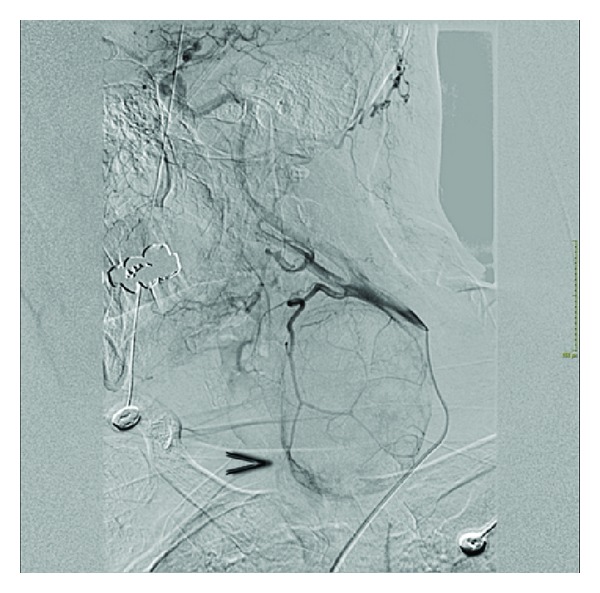
Angiography: the common carotid artery appears pervious, but displaced laterally to the presence of the hematoma at the left laterocervical level. We appreciate a haemorrhagic suffusion in terms of the superior thyroid artery, on the medial side of the hematoma (arrow).

This test revealed bleeding from the final arterial branch of the STA with extravasation (Figure 1). An attempt to embolize the final arterial branch of the STA failed, afterwards, it was decided to explore the neck surgically. Intubation was extremely difficult and foresaw the use of flexible nasopharyngoscopy. A large hematoma was found out both inside and behind the left thyroid lobe; however, no other bleeding was found out in the lateral neck compartment. Left lobectomy was performed without tracheotomy. Blood transfusions were not necessary. The pathological examination showed a widespread intraparenchymal hemorrhage and a hyperplastic adenomatous nodule. The Pathologist found out a marked wall thinning and ectasia of some branches of the STA.

## 3. Case Report Number 2

A 74-year-old woman with a small cell lung cancer that metastasized to the kidney, liver, and bones, was admitted to the internal medicine department of our hospital. The Patient presented enlarging medial cervical mass developed in four days, with progressive impairment of the upper airway. She has been suffering from Graves' disease for years.

**Figure 2:**
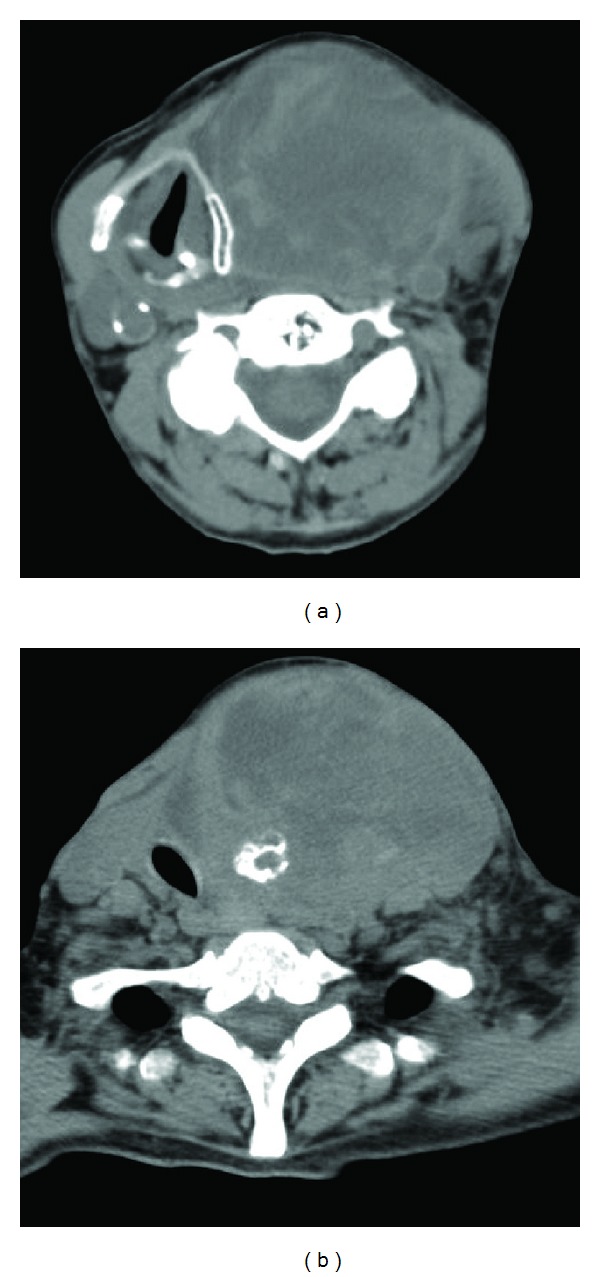
(a) Axial CT image showing the displacement of the larynx to the right by a heterogenous swelling on the left; (b) displacement of the trachea to the right by the lesion with internal calcification.

The neck computed tomography scans showed a large mass of 96 × 62 × 77 mm that originated in the left lobe of the thyroid gland with severe displacement of tracheal axis to the right side and compression of oropharynx, with impairment of upper airway lumen. Moreover, the hyoid bone and larynx turned out to be moved to more than 3 cm from the midline. Finally, a compression of the common carotid artery came out, with several nodes in all left cervical levels (Figure 2).

**Figure 3:**
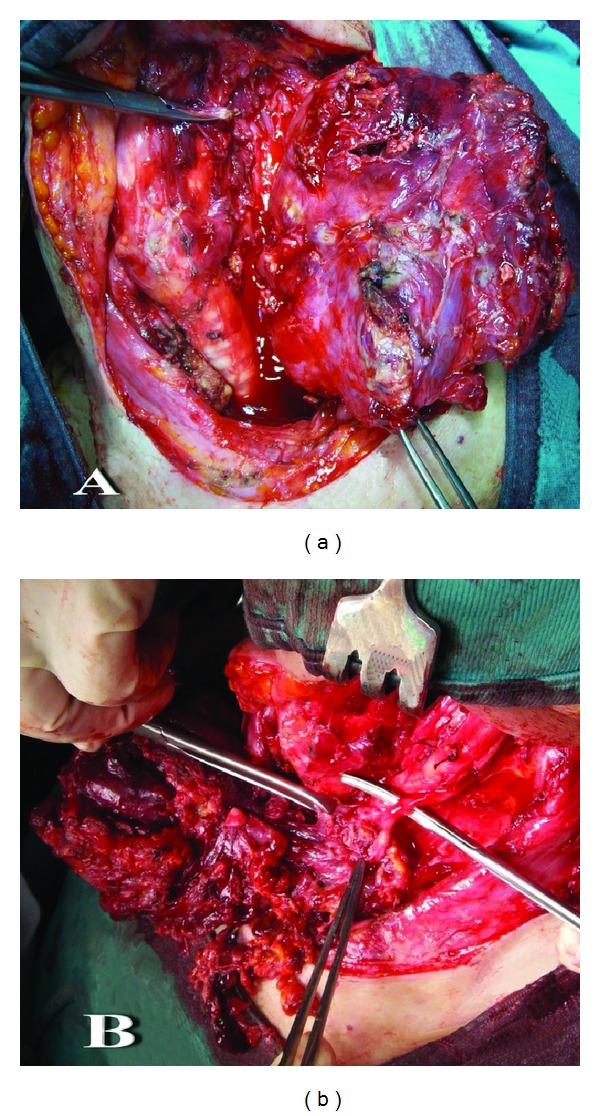
(a) Intraoperative image of the tumor originating from the left thyroideal lobe stuffed of blood; (b) the left vagus nerve, completely embedded by the tumor.

Consequently, because of an increasing dyspnea associated with dysphagia and dysphonia, a surgical exploration of her neck was carried out in emergency regimen. After a difficult oropharyngeal intubation, a large bleeding thyroid mass of the left lobe was detected. The mass spread all through the surrounding structures. Afterwards, a left hemithyroidectomy was performed with binding and resection of the left internal jugular vein, as well as the sacrifice of the left vagus nerve, completely embedded by the tumor (Figure 3). A huge quantity of limph nodes looking like malignant were present at all left cervical levels. The surgery was concluded, considering the general clinical condition of the patient and the impossibility to achieve a radical surgery. No tracheotomy was needed. Two units of packed red blood cells were transfused. The postoperative CT revealed a repositioning of the tracheal axis in the middle, with good saturation, without oxygen therapy. Histopathological examination revealed an ATC with infiltration of surrounding tissues, associated with internal bleeding.

## 4. Discussion

The cervical masses caused by a spontaneous intrathyroid haemorrhage or by a bleeding ATC are extremely rare, but also life-threatening conditions. Consequently it is important to know how to recognize them promptly, in order to prevent fatal acute airway distress [2, 3, 5, 6, 11, 12]. The only case in the literature describing a spontaneous rupture of the STA has been reported by Stenner and coworkers [13].

Because of the rarity of these entities, a wrong management may be common: the first and most important step is to secure airway, then after this, the diagnostic workup could be undertaken. Endotracheal intubation is generally used; however, sometimes nasotracheal intubation through the use of flexible fiber optic is fundamental. If it is not possible, then you could appeal to emergency tracheotomy.

Referring to the first patient, the angiography was very important. In fact, it gave us the opportunity to make a diagnosis, and secondly because it is a useful instrument to close a bleeding vessel, often representing a good alternative to open surgery.

About our second case, it is interesting to note that performing a tracheotomy could be very difficult, because of compression by the bleeding mass, that deviated tracheal axis so markedly (Figure 2). In our opinion, tracheotomy should be a second option in case of failure of guided intubation [5]. We recommend not to waste precious time, and to secure the airway when there is suspicion of its severe impairment. Tashima et al. [8] report that dyspnea at its outbreak is the only significant independent prognostic factor affecting the mortality of ATC. Following Kumar and Joshi's [14] point of view, we assert that a partial or, when it is possible to get a radical surgery, a total thyroidectomy is a possible and life-saving option for palliation of a severe compressive ATC.
